# Supplementary material for: Forb diversity globally is harmed by nutrient enrichment but can be rescued by large mammalian herbivory
Source: Commun Biol. 2025 Mar 15;8:444. doi: 10.1038/s42003-025-07882-7 (PMC11910660; doi:10.1038/s42003-025-07882-7)
Supplement: Supplementary file 2 — Supplementary Information [file 42003_2025_7882_MOESM2_ESM.pdf]

Supplemental Methods

To further account for differences in control vs treatment data, we used the method from Lind et al. 2017 *Ecology Letters*, to calculate a difference in Log Response Ratio (LRR) between the LRRs for the control and treatment data for a given site, plot and year for each response variable:

$$Difference\ in\ LRR = \left(\frac{ln(Treatment\ Response\ Variable_{Post-Treatment})}{ln(Treatment\ Response\ Variable_{Pre-Treatment}}\right) - \frac{ln(Control\ Response\ Variable_{Post-Treatment})}{ln(Control\ Response\ Variable_{Pre-Treatment}})$$

For the control treatment, the difference in LRR is 0. We then ran linear mixed models with a Gaussian distribution with our response variables as difference in LRR and the same fixed effects as described in the paper and a random effect of block nested within site to test.

Supplemental Figures & Tables

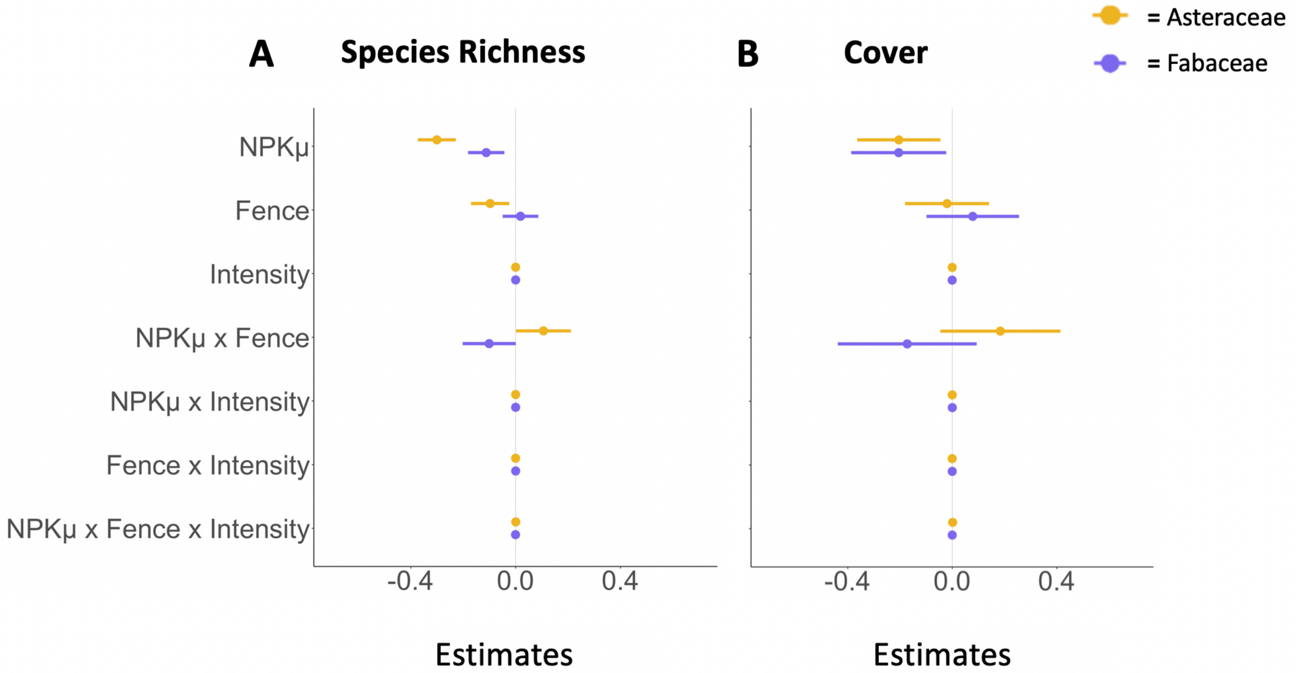

Figure S1. The effect of the fencing x fertilization experiment and herbivore intensity on (A) Species Richness and (B) Cover for Asteraceae (yellow) and Fabaceae (indigo). Model estimates of log response ratios for the effect of different treatments are shown relative to the control treatment (estimate =0). Binary response variables were converted to log response variables to account for the change from pre-treatment to current data and cover data were normalized

relative to maximum plot cover. Fence refers to herbivore exclusion fencing. NPKμ refers to the nitrogen, phosphorus and potassium with micronutrients treatment.

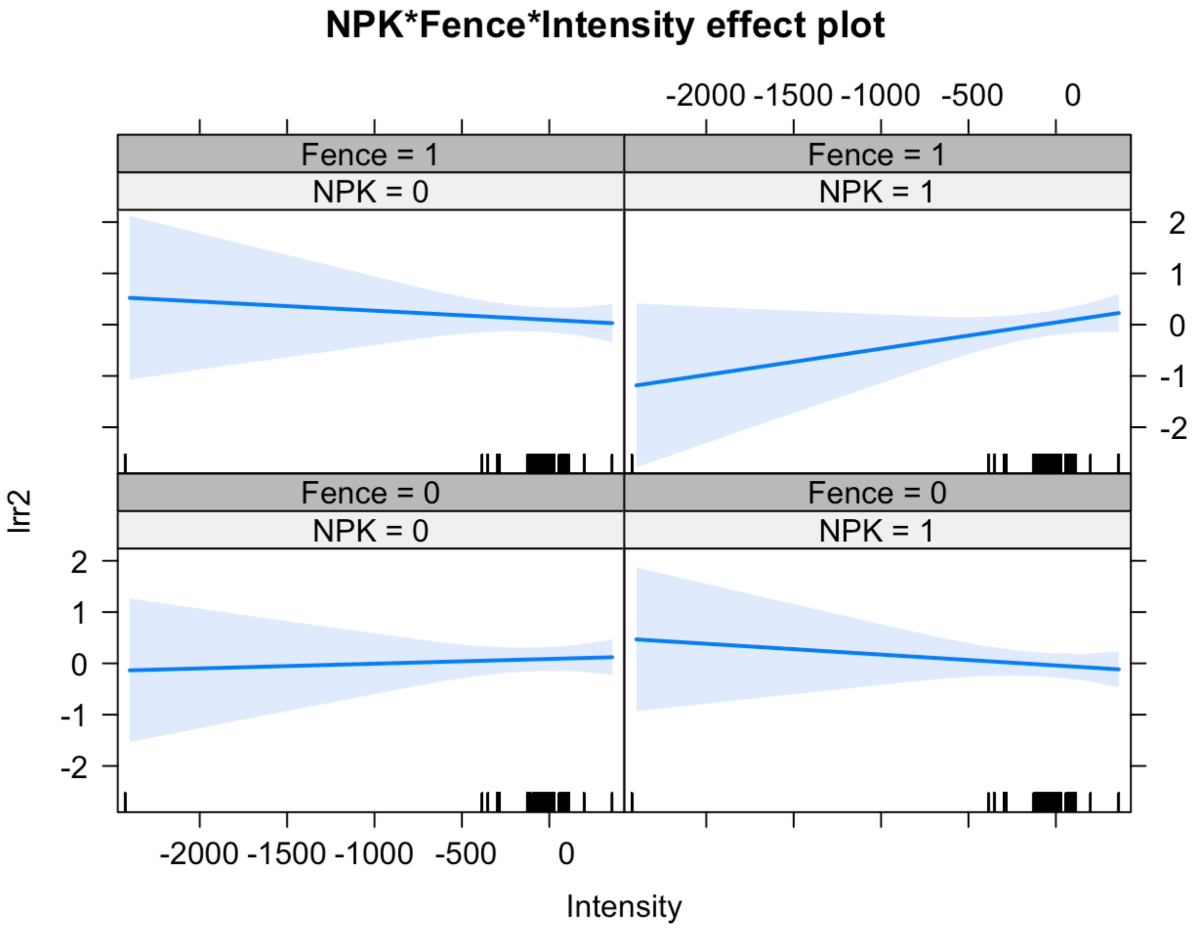

1,2

Figure S2. Three-way-interaction plots for the effect of fencing, NPKμ fertilization, and herbivore intensity on forb cover log response ratio. A more negative value for herbivore intensity corresponds to greater herbivore intensity.

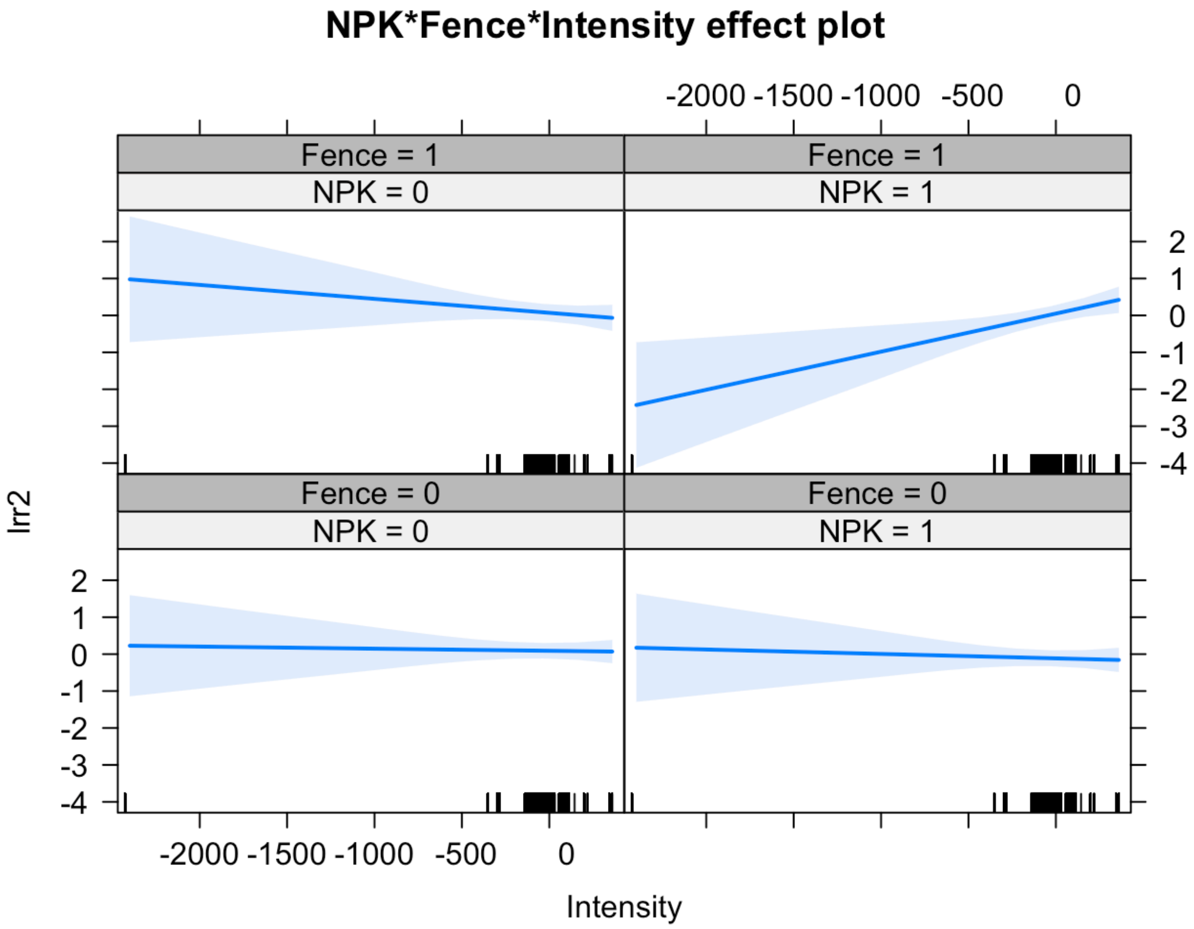

Figure S3. Three-way-interaction plots for the effect of fencing, NPKμ fertilization, and herbivore intensity on Asteraceae cover log response ratio. A more negative value for herbivore intensity corresponds to greater herbivore intensity.

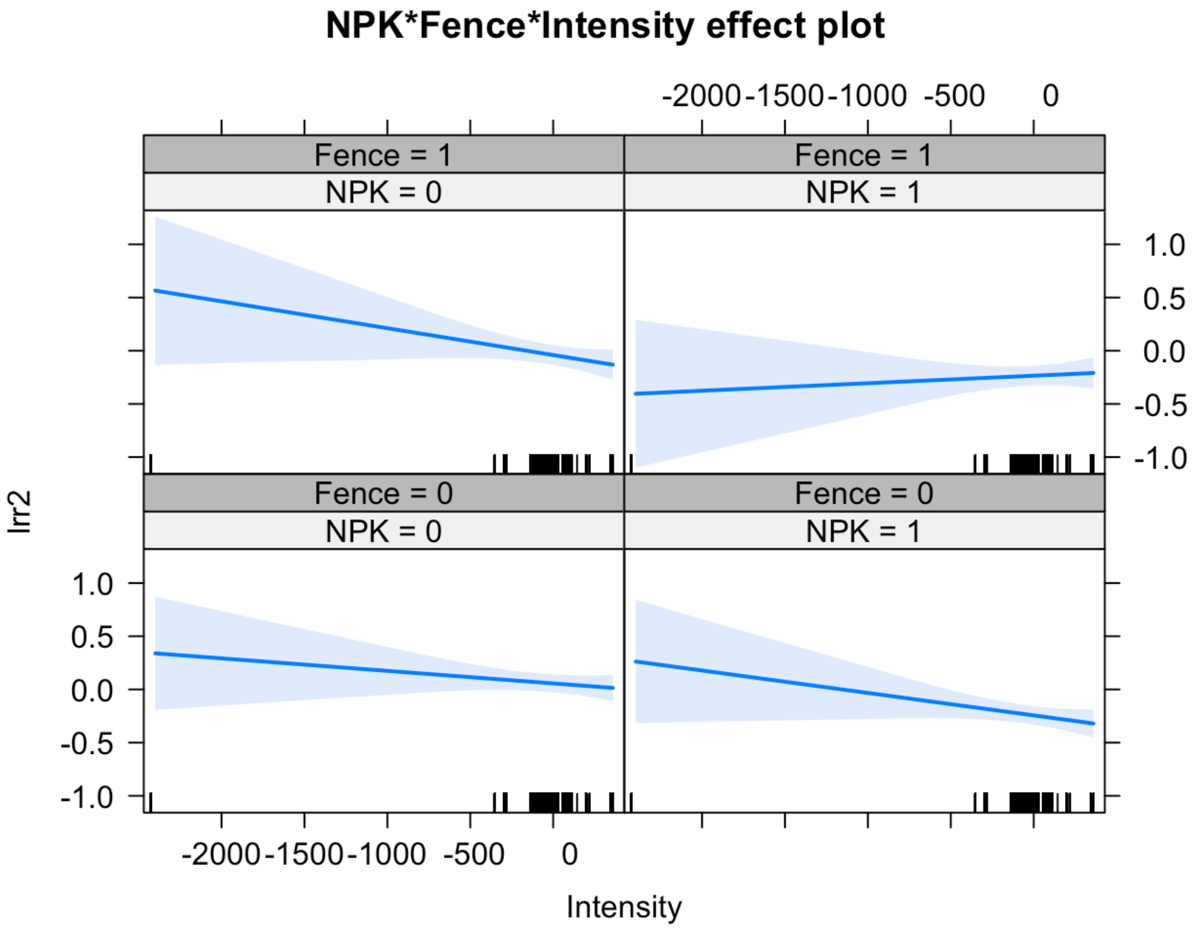

Figure S4. Three-way-interaction plots for the effect of fencing, NPKμ fertilization, and herbivore intensity on Asteraceae richness log response ratio. A more negative value for herbivore intensity corresponds to greater herbivore intensity.

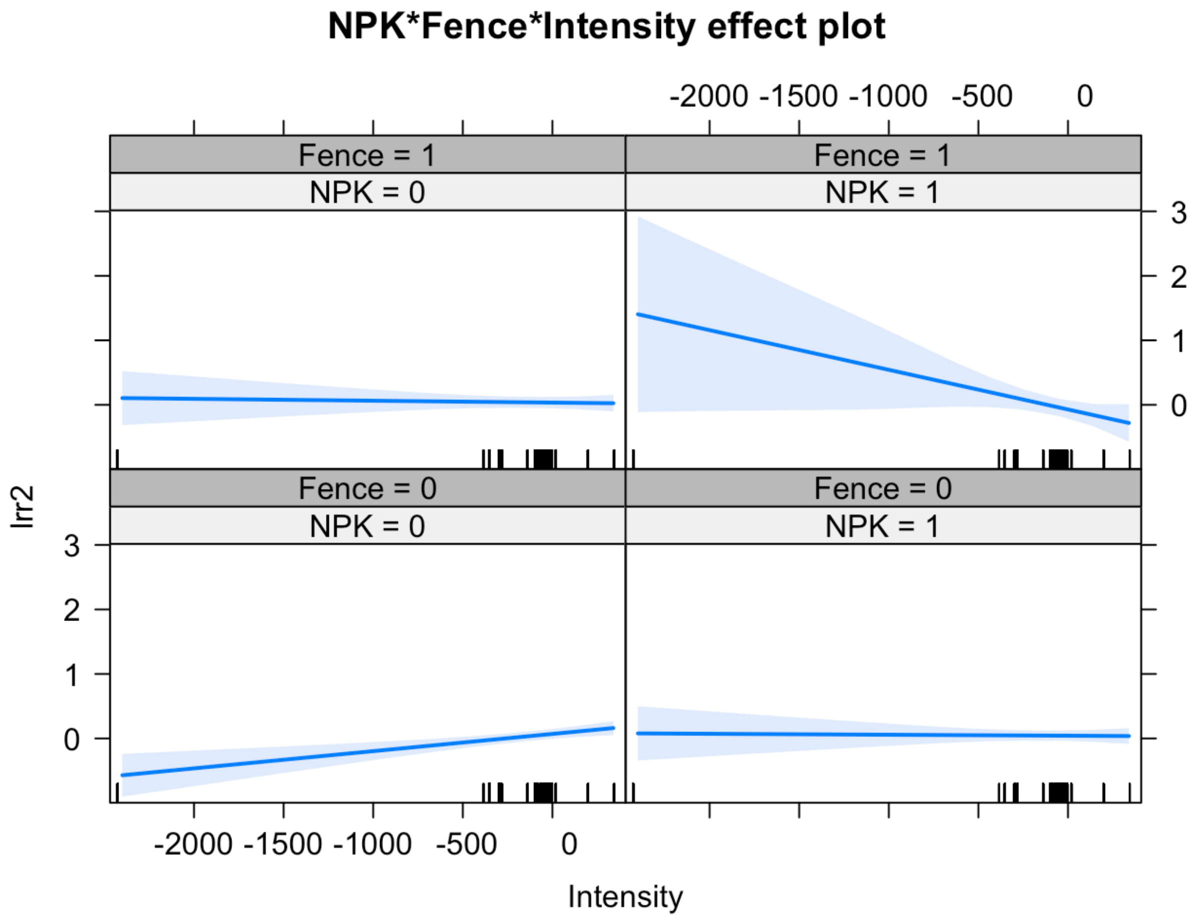

Figure S5. Three-way-interaction plots for the effect of fencing, NPKμ fertilization, and herbivore intensity on Polygonaceae richness log response ratio. A more negative value for herbivore intensity corresponds to greater herbivore intensity.

Table S1. Mixed effects model results for the effects of fertilization by nutrient type on Forb Normalized Forb Cover and Normalized Grass Cover with full interactions. The intercept is the mean value of the unfenced and unfertilized control plots. All response variables were calculated using LRRs. The parenthetical numbers are the confidence interval. * $p<0.05$ ** $p<0.01$ *** $p<0.001$

	Forb Cover			Grass Cover		
Predictors	*Estimates*	*T-value*	*p*	*Estimates*	*T-value*	*p*
**Intercept**	0.03 (-0.13 – 0.18)	0.33	0.742	-0.04 (-0.15 – 0.07)	-0.63	0.530
**N**	-0.01 (-0.10 – 0.08)	-0.14	0.888	0.07 (-0.00 – 0.14)	1.90	0.057
**P**	-0.01 (-0.10 – 0.08)	-0.19	0.848	0.10 ** (0.03 – 0.17)	2.80	**0.005**
**Kμ**	0.00 (-0.09 – 0.09)	0.05	0.962	-0.02 (-0.09 – 0.06)	-0.45	0.656
**N × P**	-0.07 (-0.20 – 0.06)	-1.10	0.271	0.02 (-0.08 – 0.13)	0.45	0.655
**N × Kμ**	-0.08 (-0.21 – 0.05)	-1.24	0.214	0.04 (-0.07 – 0.14)	0.67	0.500
**P × Kμ**	0.05 (-0.08 – 0.18)	0.80	0.424	0.00 (-0.10 – 0.10)	0.03	0.979
**N × P × Kμ**	0.04 (-0.15 – 0.22)	0.40	0.690	-0.07 (-0.21 – 0.08)	-0.88	0.379
Random Effects						
**σ^2**	0.86			0.49		
**τ_{00}**	0.63 $_{block}$			0.45 $_{block}$		
	0.25 $_{site\_code}$			0.23 $_{site\_code}$		
**N**	6 $_{block}$			6 $_{block}$		
	84 $_{site\_code}$			87 $_{site\_code}$		
**Observations**	13686			14259		

		* p<0.05	** p<0.01	*** p<0.001

Table S2. Model outputs for the effects of the fertilization factorial experiment on Difference in LRR between treatment and control.

	Forb Species Richness		***Forb Family Richness***		***Forb Cover***		***Grass Species Richness***		***Grass Cover***	
Predictors	*Estimates*	*t-value; p-value*	*Estimates*	*t-value; p-value*	*Estimates*	*t-value; p-value*	*Estimates*	*t-value; p-value*	*Estimates*	*t-value; p-value*
Intercept	-0.00 (-0.04 – 0.04)	-0.07; 0.945	-0.00 (-0.04 – 0.04)	-0.20; 0.841	-0.01 (-0.10 – 0.07)	-0.33; 0.744	0.00 (-0.03 – 0.03)	0.06; 0.956	0.04 (-0.03 – 0.10)	1.08; 0.282
N	-0.15 *** (-0.19 – -0.12)	-9.26; **<0.001**	-0.07 *** (-0.10 – -0.04)	-4.60; **<0.001**	-0.07 *** (-0.10 – -0.04)	-4.27; **<0.001**	-0.01 (-0.04 – 0.02)	-0.48; 0.628	0.08 *** (0.05 – 0.10)	6.52; **<0.001**
P	-0.07 *** (-0.10 – -0.03)	-3.98; **<0.001**	-0.02 (-0.05 – 0.01)	-1.54; 0.123	-0.03 * (-0.06 – -0.00)	-2.06; **0.039**	-0.02 (-0.05 – 0.01)	-1.49; 0.135	0.08 *** (0.06 – 0.10)	6.88; **<0.001**
Kμ	-0.04 ** (-0.08 – -0.01)	-2.70; **0.007**	-0.04 * (-0.07 – -0.01)	-2.38; **0.017**	0.03 (-0.00 – 0.06)	1.81; 0.070	-0.01 (-0.04 – 0.02)	-0.78; 0.436	-0.03 ** (-0.05 – -0.01)	-2.64; **0.008**
N × P	0.00 (-0.04 – 0.05)	0.13; 0.896	-0.04 (-0.08 – 0.01)	t=-1.60; p=0.109			0.03 (-0.01 – 0.07)	1.53; 0.126		
N × Kμ	0.03 (-0.02 – 0.07)	1.09; 0.278	0.04 (-0.00 – 0.09)	t=1.93; p=0.054			0.02 (-0.02 – 0.06)	0.87; 0.382		
P × Kμ	0.01 (-0.04 – 0.05)	0.25; 0.805	-0.03 (-0.07 – 0.02)	-1.18; 0.237			0.06 ** (0.01 – 0.10)	2.68; **0.007**		
N × P × Kμ	-0.03 (-0.09 – 0.04)	-0.74; 0.457	-0.03 (-0.09 – 0.03)	-0.97; 0.333			-0.18 *** (-0.24 – -0.12)	-6.21; **<0.001**		
Random Effects										
σ^2	0.29		0.26		1.00		0.22		0.58	
τ_{00}	0.07_{block}		0.05_{block}		0.25_{block}		0.02_{block}		0.18_{block}	
	0.27_{site_code}		0.26_{site_code}		0.50_{site_code}		0.23_{site_code}		0.39_{site_code}	
ICC	0.54		0.55		0.43		0.53		0.49	
N	6_{block}		6_{block}		6_{block}		6_{block}		6_{block}	

	84 site_code	84 site_code	84 site_code	87 site_code	87 site_code
Observations	16447	16251	16809	16093	17742

* *p<0.05* ** *p<0.01* *** *p<0.001*

Table S3. Statistical output for type III ANOVA significance testing for mixed effects model results for the effects of fertilization by nutrient type on Forb Family Richness, Forb Species Richness, Normalized Forb Cover, Grass Species Richness and Normalized Grass Cover. The intercept is the mean value of the unfenced and unfertilized control plots. All response variables were calculated using LRRs. The alpha level for statistical significance is 0.05, and for all parameters df=1.

	**Forb Species Richness**		**Forb Family Richness**		**Forb Cover**		**Grass Species Richness**		**Grass Cover**	
**Predictors**	*Chi-Squared Value*	*P-value*	*Chi-Squared Value*	*P-value*	*Chi-Squared Value*	*P-value*	*Chi-Squared Value*	*P-value*	*Chi-Squared Value*	*P-value*
**Intercept**	0.1448	0.70	0.0360	0.85	0.2835	0.60	0.1602	0.69	0.4036	0.53
**N**	**37.4728**	**<0.0001**	**12.1493**	**0.00049**	**10.1370**	**0.00145**	0.4480	0.53	**19.6752**	**<0.0001**
**P**	**5.3792**	**0.0204**	0.2879	0.5915496	0.1331	0.72	0.3452	0.56	**27.9382**	**<0.0001**
**Kμ**	**6.7188**	**0.0095**	3.1615	0.075	0.0098	0.92	0.0125	0.91	0.6008	0.44
**N × P**	0.0038	0.95	0.6477	0.42			0.3545	0.55		
**N × Kμ**	1.9240	0.17	2.0812	0.15			0.0001	0.99		
**P × Kμ**	0.3761	0.54	0.6940	0.40			1.6140	0.20		
**N × P × Kμ**	1.1440	0.28	0.4553	0.50			**9.5834**	**0.0020**		

Table S4. Effects of fertilization on cover and richness of key floral families. Mixed effects model results for the effects of fertilization by nutrient type on Asteraceae Richness, Asteraceae Cover, Fabaceae Richness, Fabaceae Cover, Geraniaceae Richness, Geraniaceae Cover, Apiaceae Richness, Apiaceae Cover, Polygonaceae Richness, Polygonaceae Cover. All response variables were calculated using LRRs. The intercept is the mean value of the unfenced and unfertilized control plots. The parenthetical numbers are the confidence interval. * p<0.05 ** p<0.01 *** p<0.001

	Asteraceae Richness		Asteraceae Cover		Fabaceae Richness		Fabaceae Cover		Geraniaceae Richness		Geraniaceae Cover	
Predictors	*Estimates*	*t-value; p-value*	*Estimates*	*t-value; p-value*	*Estimates*	*t-value; p-value*	*Estimates*	*t-value; p-value*	*Estimates*	*t-value; p-value*	*Estimates*	*t-value; p-value*
**Intercept**	0.05 (-0.02 – 0.11)	1.36; 0.174	0.05 (-0.11 – 0.20)	0.56; 0.574	-0.04 (-0.09 – 0.02)	-1.27; 0.204	0.20 * (0.01 – 0.40)	**2.09; 0.037**	0.07 (-0.01 – 0.15)	1.64; 0.101	0.19 (-0.27 – 0.66)	0.81; 0.417
**N**	-0.14 *** (-0.20 – -0.08)	**-4.49; <0.001**	-0.04 (-0.11 – 0.03)	-1.13; 0.258	-0.05 (-0.11 – 0.01)	-1.53; 0.127	-0.56 *** (-0.65 – -0.47)	**-11.90; <0.001**	0.03 (-0.04 – 0.09)	0.84; 0.401	0.03 (-0.16 – 0.22)	0.33; 0.739
**P**	-0.12 *** (-0.18 – -0.06)	**-3.96; <0.001**	-0.07 * (-0.14 – -0.01)	**-2.15; 0.032**	0.08 ** (0.02 – 0.14)	**2.58; 0.010**	0.17 *** (0.08 – 0.26)	**3.61; <0.001**	-0.10 ** (-0.16 – -0.04)	**-3.17; 0.002**	0.12 (-0.07 – 0.32)	1.28; 0.199
**Kμ**	-0.06 * (-0.12 – -0.01)	**-2.13; 0.033**	-0.07 * (-0.14 – -0.00)	**-1.99; 0.046**	0.02 (-0.04 – 0.08)	0.66; 0.508	0.15 ** (0.06 – 0.24)	**3.23; 0.001**	-0.00 (-0.06 – 0.06)	-0.13; 0.900	-0.32 *** (-0.52 – -0.13)	**-3.32; 0.001**
**N × P**	0.07 (-0.01 – 0.16)	1.62; 0.106			-0.09 * (-0.18 – -0.00)	**-1.98; 0.047**			0.07 (-0.02 – 0.16)	1.44; 0.149		
**N × Kμ**	0.00 (-0.08 – 0.09)	0.04; 0.971			-0.07 (-0.16 – 0.02)	-1.57; 0.116			-0.04 (-0.13 – 0.05)	-0.85; 0.395		
**P × Kμ**	0.04 (-0.05 – 0.12)	0.82; 0.410			-0.04 (-0.12 – 0.05)	-0.80; 0.424			0.09 (-0.00 – 0.18)	1.85; 0.064		
**N × P × Kμ**	-0.02 (-0.14 – 0.10)	-0.35; 0.726			0.06 (-0.07 – 0.19)	0.93; 0.354			-0.06 (-0.20 – 0.07)	-0.97; 0.332		

Random Effects						
σ^2	0.26	1.23	0.17	1.54	0.07	2.01
τ_{00}	0.22 block	0.60 block	0.13 block	0.66 block	0.14 block	0.93 block

	0.13 site_code	0.34 site_code	0.10 site_code	0.30 site_code	0.08 site_code	0.24 site_code
**N**	6 block	6 block	6 block	6 block	5 block	5 block
	81 site_code	81 site_code	70 site_code	70 site_code	21 site_code	21 site_code
**Observations**	10951	10951	5900	5900	1774	1774

	**Apiaceae Richness**		**Apiaceae Cover**		**Polygonaceae Richness**		**Polygonaceae Cover**	
Predictors	*Estimates*	*t-value; p-value*	*Estimates*	*t-value; p-value*	*Estimates*	*t-value; p-value*	*Estimates*	*t-value; p-value*
**Intercept**	0.02 (-0.03 – 0.07)	0.80; 0.424	0.33 * (0.03 – 0.63)	**2.18; 0.029**	0.01 (-0.05 – 0.08)	0.44; 0.663	0.13 (-0.12 – 0.37)	0.99; 0.321
**N**	-0.04 (-0.10 – 0.02)	-1.20; 0.231	-0.16 (-0.36 – 0.03)	-1.65; 0.100	-0.00 (-0.08 – 0.08)	-0.05; 0.963	0.34 *** (0.16 – 0.52)	**3.62; <0.001**
**P**	-0.00 (-0.06 – 0.06)	-0.05; 0.962	0.08 (-0.11 – 0.28)	0.85; 0.396	0.02 (-0.06 – 0.10)	0.56; 0.576	0.05 (-0.13 – 0.24)	0.58; 0.563
**Kμ**	0.04 (-0.02 – 0.10)	1.36; 0.173	0.01 (-0.19 – 0.20)	0.06; 0.956	-0.05 (-0.13 – 0.03)	-1.32; 0.189	-0.19 * (-0.38 – -0.01)	**-2.08; 0.037**
**N × P**	0.04 (-0.05 – 0.13)	0.86; 0.390			0.01 (-0.10 – 0.12)	0.15; 0.884		
**N × Kμ**	0.01 (-0.08 – 0.11)	0.32; 0.752			0.04 (-0.07 – 0.15)	0.73; 0.463		
**P × Kμ**	-0.05 (-0.14 – 0.03)	-1.20; 0.231			0.01 (-0.10 – 0.12)	0.19; 0.852		
**N × P × Kμ**	0.02 (-0.10 – 0.15)	0.36; 0.719			-0.02 (-0.18 – 0.14)	-0.21; 0.836		

Random Effects				
**σ^2**	0.05	1.76	0.10	2.06
**τ_{00}**	0.07 block	0.58 block	0.09 block	0.40 block
	0.08 site_code	0.34 site_code	0.06 site_code	0.36 site_code
**N**	5 block	5 block	5 block	5 block
	35 site_code	35 site_code	33 site_code	33 site_code
**Observations**	1888	1888	2354	2354

Table S5. Effects of fertilization on cover of key floral families with full interactions. Mixed effects model results for the effects of fertilization by nutrient type on Asteraceae Cover, Fabaceae Cover, Geraniaceae Cover, Apiaceae Cover, Polygonaceae Cover. All response variables were calculated using LRRs. The intercept is the mean value of the unfenced and unfertilized control plots. The parenthetical numbers are the confidence interval. * p<0.05   ** p<0.01   *** p<0.001

	Asteraceae Cover			Fabaceae Cover			Geraniaceae Cover			Apiaceae Cover			Polygonaceae Cover		
Predictors	*Estimates*	*T-value*	*p*	*Estimates*	*T-value*	*p*	*Estimates*	*T-value*	*p*	*Estimates*	*T-value*	*p*	*Estimates*	*T-value*	*p*
**Intercept**	0.04 (-0.13 – 0.21)	0.50	0.617	0.10 (-0.11 – 0.31)	0.92	0.357	0.08 (-0.41 – 0.56)	0.31	0.760	0.27 (-0.06 – 0.61)	1.61	0.108	-0.07 (-0.37 – 0.23)	-0.46	0.648
**N**	-0.01 (-0.14 – 0.12)	-0.16	0.873	-0.34 *** (-0.51 – -0.16)	-3.66	**<0.001**	0.29 (-0.07 – 0.65)	1.57	0.116	-0.02 (-0.41 – 0.37)	-0.10	0.924	0.41 * (0.05 – 0.78)	2.22	**0.026**
**P**	-0.10 (-0.24 – 0.03)	-1.53	0.125	0.37 *** (0.19 – 0.55)	4.12	**<0.001**	0.18 (-0.19 – 0.54)	0.95	0.340	0.16 (-0.19 – 0.51)	0.90	0.369	0.44 * (0.09 – 0.80)	2.44	**0.015**
**Kμ**	-0.00 (-0.14 – 0.13)	-0.06	0.952	0.22 * (0.04 – 0.39)	2.45	**0.014**	-0.12 (-0.48 – 0.24)	-0.66	0.509	0.25 (-0.13 – 0.62)	1.28	0.202	0.10 (-0.25 – 0.45)	0.57	0.566
**N × P**	0.00 (-0.19 – 0.20)	0.05	0.961	-0.41 ** (-0.67 – -0.15)	-3.12	**0.002**	-0.06 (-0.59 – 0.47)	-0.24	0.813	-0.17 (-0.73 – 0.38)	-0.62	0.536	-0.14 (-0.65 – 0.37)	-0.53	0.594
**N × Kμ**	-0.19 (-0.38 – 0.00)	-1.93	0.053	-0.13 (-0.38 – 0.13)	-0.97	0.332	-0.38 (-0.90 – 0.15)	-1.40	0.162	-0.48 (-1.05 – 0.08)	-1.67	0.095	0.05 (-0.47 – 0.56)	0.17	0.862
**P × Kμ**	-0.07 (-0.26 – 0.12)	-0.73	0.465	-0.09 (-0.34 – 0.16)	-0.70	0.481	0.05 (-0.48 – 0.58)	0.18	0.854	-0.36 (-0.89 – 0.17)	-1.32	0.186	-0.59 * (-1.09 – -0.09)	-2.30	**0.021**
**N × P × Kμ**	0.25 (-0.02 – 0.53)	1.82	0.069	0.16 (-0.21 – 0.52)	0.84	0.403	-0.21 (-0.97 – 0.56)	-0.54	0.592	0.70 (-0.09 – 1.49)	1.73	0.084	-0.12 (-0.84 – 0.61)	-0.31	0.754
Random Effects															
σ^2	1.23			1.54			2.00			1.76			2.03		
τ_{00}	0.60 $_{block}$			0.66 $_{block}$			0.93 $_{block}$			0.58 $_{block}$			0.40 $_{block}$		
	0.33 $_{site\_code}$			0.30 $_{site\_code}$			0.25 $_{site\_code}$			0.34 $_{site\_code}$			0.36 $_{site\_code}$		
N	6 $_{block}$			6 $_{block}$			5 $_{block}$			5 $_{block}$			5 $_{block}$		
	81 $_{site\_code}$			70 $_{site\_code}$			21 $_{site\_code}$			35 $_{site\_code}$			33 $_{site\_code}$		
Observations	10951			5900			1774			1888			2354		
													** p<0.05 ** p<0.01 *** p<0.001*		

Table S6. Statistical output for type III ANOVA significance testing for effects of fertilization on cover and richness of key floral families. Mixed effects model results for the effects of fertilization by nutrient type on Asteraceae Richness, Asteraceae Cover, Fabaceae Richness, Fabaceae Cover, Geraniaceae Richness, Geraniaceae Cover, Apiaceae Richness, Apiaceae Cover, Polygonaceae Richness, Polygonaceae Cover. All response variables were calculated using LRRs. The intercept is the mean value of the unfenced and unfertilized control plots. The alpha level for statistical significance is 0.05, and for all parameters df=1.

	**Asteraceae Richness**		**Asteraceae Cover**		**Fabaceae Richness**		**Fabaceae Cover**		**Geraniaceae Richness**		**Geraniaceae Cover**	
Predictors	*Chi-Squared Value*	*P-value*	*Chi-Squared Value*	*P-value*	*Chi-Squared Value*	*P-value*	*Chi-Squared Value*	*P-value*	*Chi-Squared Value*	*P-value*	*Chi-Squared Value*	*P-value*
**Intercept**	1.8516	0.17	0.3164	0.57	1.6166	0.20	4.350	0.037	2.6978	0.10	0.6590	0.42
**N**	**20.2007**	**<0.0001**	1.2811	0.26	2.3318	0.13	**141.708**	**<0.0001**	0.7053	0.40	0.1111	0.74
**P**	**15.6892**	**<0.0001**	**4.6086**	**0.032**	**6.6748**	**0.0098**	**13.044**	**0.0003**	**10.0207**	**0.0015**	1.6491	0.20
**Kμ**	**4.5537**	**0.033**	**3.9660**	**0.046**	0.4376	0.51	**10.425**	**0.0012**	0.0159	0.90	**11.0505**	**0.00089**
**N × P**	2.6143	0.11			3.9326	0.0474			2.0797	0.15		
**N × Kμ**	0.0013	0.97			2.4757	0.12			0.7224	0.40		
**P × Kμ**	0.6781	0.41			0.6381	0.42			3.4353	0.064		
**N × P × Kμ**	0.1231	0.73			0.8586	0.35			0.9428	0.33		

	**Apiaceae Richness**		**Apiaceae Cover**		**Polygonaceae Richness**		**Polygonaceae Cover**	
Predictors	*Chi-Squared Value*	*P-value*	*Chi-Squared Value*	*P-value*	*Chi-Squared Value*	*P-value*	*Chi-Squared Value*	*P-value*
**Intercept**	0.6383	0.42	4.7576	0.03	0.1905	0.66	0.9863	0.32
**N**	1.4358	0.23	2.7073	0.10	0.0021	0.96	**13.1108**	**0.00029**
**P**	0.0022	0.96	0.7213	0.40	0.3121	0.58	0.3340	0.56
**Kμ**	1.8545	0.17	0.0031	0.96	1.7292	0.19	**4.3331**	**0.037**
**N × P**	0.7396	0.39			0.0212	0.88		
**N × Kμ**	0.0996	0.75			0.5394	0.46		
**P × Kμ**	1.4376	0.23			0.0346	0.85		
**N × P × Kμ**	0.1290	0.72			0.0431	0.84		

Table S7. Model outputs for the effects of the fencing by fertilization experiment on Difference in LRR between treatment and control.

	**Forb Species Richness**		**Forb Family Richness**		**Forb Cover**		**Grass Species Richness**		**Grass Cover**	
Predictors	*Estimates*	*t-value; p-value*	*Estimates*	*t-value; p-value*	*Estimates*	*t-value; p-value*	*Estimates*	*t-value; p-value*	*Estimates*	*t-value; p-value*
**Intercept**	-0.01<br>(-0.07 – 0.05)	-0.33<br>0.743	-0.02<br>(-0.08 – 0.04)	-0.74<br>0.458	-0.07<br>(-0.21 – 0.07)	-0.97<br>0.331	0.00<br>(-0.04 – 0.04)	0.11<br>0.915	0.05<br>(-0.07 – 0.16)	0.81<br>0.416
**NPKμ**	-0.32 ***<br>(-0.36 – -0.27)	-13.31<br>**<0.001**	-0.20 ***<br>(-0.24 – -0.15)	-8.73<br>**<0.001**	-0.14 **<br>(-0.22 – -0.05)	-3.12<br>**0.002**	-0.09 ***<br>(-0.12 – -0.05)	-4.55<br>**<0.001**	0.20 ***<br>(0.13 – 0.27)	5.42<br>**<0.001**
**Fence**	-0.06 *<br>(-0.10 – -0.01)	-2.41<br>**0.016**	0.02<br>(-0.02 – 0.07)	1.03<br>0.305	0.00<br>(-0.08 – 0.09)	0.06<br>0.954	0.02<br>(-0.02 – 0.06)	1.17<br>0.240	0.08 *<br>(0.01 – 0.15)	2.22<br>**0.026**
**PET s**	-0.01<br>(-0.07 – 0.05)	-0.30<br>0.762	-0.02<br>(-0.07 – 0.04)	-0.61<br>0.542	-0.09<br>(-0.21 – 0.04)	-1.36<br>0.173	0.01<br>(-0.03 – 0.05)	0.39<br>0.693	0.01<br>(-0.08 – 0.10)	0.22<br>0.822
**Intensity**	-0.00<br>(-0.00 – 0.00)	-0.16<br>0.872	-0.00<br>(-0.00 – 0.00)	-0.09<br>0.928	0.00<br>(-0.00 – 0.00)	0.27<br>0.786	-0.00<br>(-0.00 – 0.00)	-0.26<br>0.793	-0.00<br>(-0.00 – 0.00)	-0.19<br>0.853
**NPKμ × Fence**	0.05<br>(-0.01 – 0.12)	1.57<br>0.115	0.02<br>(-0.05 – 0.08)	0.54<br>0.588	0.10<br>(-0.03 – 0.22)	1.55<br>0.122	-0.08 **<br>(-0.13 – -0.02)	-2.87<br>**0.004**	-0.16 **<br>(-0.26 – -0.06)	-3.09<br>**0.002**
**NPKμ × PET s**	-0.01<br>(-0.06 – 0.05)	-0.24<br>0.810	0.01<br>(-0.04 – 0.06)	0.41<br>0.680	-0.08<br>(-0.18 – 0.01)	-1.67<br>0.094	-0.03<br>(-0.07 – 0.01)	-1.42<br>0.156	-0.01<br>(-0.09 – 0.06)	-0.27<br>0.785
**NPKμ × Intensity**	-0.00 **<br>(-0.00 – -0.00)	-3.13<br>**0.002**	-0.00<br>(-0.00 – 0.00)	-1.34<br>0.181	-0.00 **<br>(-0.00 – -0.00)	-2.92<br>**0.003**	0.00<br>(-0.00 – 0.00)	1.01<br>0.315	0.00<br>(-0.00 – 0.00)	0.19<br>0.848
**Fence × PET s**	-0.07 *<br>(-0.12 – -0.01)	-2.50<br>**0.012**	-0.06 *<br>(-0.11 – -0.01)	-2.50<br>**0.012**	-0.05<br>(-0.15 – 0.04)	-1.12<br>0.265	-0.05 *<br>(-0.09 – -0.01)	-2.55<br>**0.011**	0.06<br>(-0.02 – 0.13)	1.51<br>0.130
**Fence × Intensity**	0.00<br>(-0.00 – 0.00)	0.83<br>0.405	0.00 ***<br>(0.00 – 0.00)	3.34<br>**0.001**	-0.00<br>(-0.00 – 0.00)	-1.73<br>0.085	0.00<br>(-0.00 – 0.00)	1.82<br>0.069	0.00 *<br>(0.00 – 0.00)	2.38<br>**0.017**
**NPKμ × Fence × PET s**	-0.02<br>(-0.10 – 0.05)	-0.63<br>0.530	0.05<br>(-0.02 – 0.12)	1.29<br>0.196	0.01<br>(-0.12 – 0.15)	0.20<br>0.840	-0.04<br>(-0.10 – 0.02)	-1.34<br>0.180	-0.11 *<br>(-0.22 – -0.00)	-2.03<br>**0.042**
**NPKμ × Fence × Intensity**	0.00<br>(-0.00 – 0.00)	1.27<br>0.203	-0.00 *<br>(-0.00 – -0.00)	-2.16<br>**0.031**	0.00 ***<br>(0.00 – 0.00)	5.10<br>**<0.001**	0.00<br>(-0.00 – 0.00)	0.32<br>0.752	-0.00 **<br>(-0.00 – -0.00)	-2.70<br>**0.007**
**Random Effects**										
σ^2	0.23		0.21		0.79		0.16		0.57	
τ_{00}	0.09 block		0.06 block		0.33 block		0.04 block		0.29 block	
	0.28 site_code		0.27 site_code		0.46 site_code		0.18 site_code		0.34 site_code	
**ICC**	0.61		0.61		0.50		0.58		0.53	
**N**	6 block		6 block		6 block		6 block		6 block	
	46 site_code		46 site_code		46 site_code		48 site_code		48 site_code	

**Observations**	3729	3681	3743	3648	3798

***** *p<0.05* **** *p<0.01* ***** *p<0.001***

Table S8. Statistical output for type III ANOVA significance testing for mixed effects model results for the effects of fertilization by fencing, herbivore intensity, and potential evapotranspiration (PET) on Forb Family Richness, Forb Species Richness, Normalized Forb Cover, Grass Species Richness and Normalized Grass Cover. All response variables were calculated using LRRs. The intercept is the mean value of the unfenced and unfertilized control plots. The alpha level for statistical significance is 0.05, and for all parameters df=1.

	**Forb Species Richness**		**Forb Family Richness**		**Forb Cover**		**Grass Species Richness**		**Grass Cover**	
Predictors	*Chi-Squared Value*	*P-value*	*Chi-Squared Value*	*P-value*	*Chi-Squared Value*	*P-value*	*Chi-Squared Value*	*P-value*	*Chi-Squared Value*	*P-value*
**Intercept**	0.0071	0.93	0.1493	0.70	0.5605	0.45	1.3865	0.24	2.2183	0.14
**NPKμ**	**65.7349**	**<0.0001**	**36.4746**	**<0.0001**	**4.3017**	**0.038**	**6.2600**	**0.012**	**10.7149**	**0.0011**
**Fence**	3.2502	0.071	0.0269	0.87	0.0074	0.93	1.0742	0.30	2.2554	0.13
**PET**	**11.2579**	**0.00079**	**15.0659**	**0.0001**	0.0005	.98	1.2759	0.26	1.5720	0.21
**Intensity**	0.0048	0.94	0.0186	0.089	0.0927	0.76	**6.7512**	**0.0094**	0.0345	0.85
**NPKμ × Fence**	1.4544	0.23	0.6362	0.43	0.5793	0.45	**3.9084**	**0.048**	3.6554	0.056
**NPKμ × PET**	0.0040	0.94	0.0289	0.87	1.4971	0.22	0.1750	0.68	0.3599	0.55
**NPKμ × Intensity**	**3.8487**	**0.04979**	1.0518	0.31	3.0292	0.082	0.0882	0.77	0.0234	0.88
**Fence × PET**	0.3977	0.53	0.4689	0.49	0.0135	0.91	1.0090	0.32	0.1019	0.75
**Fence × Intensity**	0.0130	0.91	2.4675	0.12	1.2858	0.26	0.4257	0.51	1.2980	0.25
**NPKμ × Fence × PET**	0.3288	0.57	0.0728	0.79	0.0132	0.91	1.1843	0.28	0.1169	0.73
**NPKμ × Fence × Intensity**	0.9111	0.34	0.5732	0.45	**9.3665**	**0.0022**	0.0510	0.82	2.6840	0.10

Table S9. Effects of fertilization by fencing and herbivore intensity on cover and richness of key floral families. Mixed effects model results for the effects of fertilization by herbivore exclusion via fencing on Asteraceae Richness, Asteraceae Cover, Fabaceae Richness, Fabaceae Cover, Geraniaceae Richness, Geraniaceae Cover, Apiaceae Richness, Apiaceae Cover, Polygonaceae Richness, Polygonaceae Cover. . The intercept is the mean value of the unfenced and unfertilized control plots. All response variables were calculated using LRRs. The parenthetical numbers are the confidence interval. * p<0.05 ** p<0.01 *** p<0.001

	**Asteraceae Richness**		**Asteraceae Cover**		**Fabaceae Richness**		**Fabaceae Cover**		**Geraniaceae Richness**		**Geraniaceae Cover**	
Predictors	*Estimates*	*t-value; p-value*	*Estimates*	*t-value; p-value*	*Estimates*	*t-value; p-value*	*Estimates*	*t-value; p-value*	*Estimates*	*t-value; p-value*	*Estimates*	*t-value; p-value*
**Intercept**	0.06 (-0.02 – 0.14)	1.40; 0.161	0.09 (-0.11 – 0.29)	0.88; 0.379	-0.02 (-0.08 – 0.05)	-0.52; 0.604	0.03 (-0.25 – 0.31)	0.22; 0.828	0.03 (-0.05 – 0.10)	0.68; 0.495	-0.06 (-0.55 – 0.43)	-0.24; 0.809
**NPKμ**	-0.30 *** (-0.37 – -0.23)	**-8.13; <0.001**	-0.20 * (-0.36 – -0.05)	**-2.52; 0.012**	-0.11 ** (-0.18 – -0.04)	**-3.18; 0.002**	-0.21 * (-0.39 – -0.02)	**-2.21; 0.027**	-0.03 (-0.11 – 0.05)	-0.80; 0.424	-0.22 (-0.63 – 0.19)	-1.06; 0.289
**Fence**	-0.10 ** (-0.17 – -0.02)	**-2.60; 0.009**	-0.02 (-0.18 – 0.14)	-0.24; 0.807	0.02 (-0.05 – 0.09)	0.53; 0.593	0.08 (-0.10 – 0.26)	0.87; 0.386	-0.01 (-0.09 – 0.06)	-0.28; 0.782	-0.17 (-0.56 – 0.22)	-0.84; 0.403
**Intensity**	-0.00 (-0.00 – 0.00)	-1.03; 0.308	-0.00 (-0.00 – 0.00)	-0.19; 0.847	0.00 (-0.00 – 0.00)	0.05; 0.962	-0.00 (-0.00 – 0.00)	-1.03; 0.308	-0.00 (-0.00 – 0.00)	-0.17; 0.871	-0.00 (-0.01 – 0.00)	-0.34; 0.740

**NPKμ × Fence**	0.11 * (0.00 – 0.21)	**1.99; 0.046**	0.18 (-0.05 – 0.41)	1.57; 0.116	-0.10 (-0.20 – 0.00)	-1.96; 0.051	-0.17 (-0.44 – 0.09)	-1.27; 0.204	-0.01 (-0.12 – 0.11)	-0.11; 0.915	0.95 ** (0.37 – 1.54)	**3.22; 0.001**
**NPKμ × Intensity**	-0.00 (-0.00 – 0.00)	-0.84; 0.402	-0.00 (-0.00 – 0.00)	-0.26; 0.799	-0.00 (-0.00 – 0.00)	-0.59; 0.554	0.00 (-0.00 – 0.00)	0.41; 0.680	-0.00 (-0.00 – 0.00)	-0.75; 0.451	0.00 (-0.00 – 0.01)	1.67; 0.094
**Fence × Intensity**	-0.00 (-0.00 – 0.00)	-0.97; 0.331	-0.00 (-0.00 – 0.00)	-1.04; 0.296	0.00 (-0.00 – 0.00)	0.64; 0.521	-0.00 (-0.00 – 0.00)	-0.53; 0.594	0.00 (-0.00 – 0.00)	0.18; 0.861	0.00 (-0.00 – 0.01)	1.41; 0.159
**NPKμ × Fence × Intensity**	0.00 * (0.00 – 0.00)	**2.14; 0.033**	0.00 *** (0.00 – 0.00)	**3.44; 0.001**	-0.00 (-0.00 – 0.00)	-1.35; 0.176	-0.00 (-0.00 – 0.00)	-0.11; 0.910	0.00 (-0.00 – 0.00)	0.18; 0.857	-0.01 (-0.01 – 0.00)	-1.62; 0.105
**σ^2**	0.27		1.26		0.17		1.35		0.07		1.89	
**τ_{00}**	0.22 block		0.60 block		0.07 block		0.78 block		0.09 block		0.72 block	
	0.15 site_code		0.41 site_code		0.18 site_code		0.64 site_code		0.07 site_code		0.49 site_code	
**N**	6 block		6 block		6 block		6 block		5 block		5 block	
	63 site_code		63 site_code		55 site_code		55 site_code		18 site_code		18 site_code	
**Observations**	4223		4223		2336		2336		778		778	

	**Apiaceae Richness**	**Apiaceae Cover**	**Polygonaceae Richness**	**Polygonaceae Cover**

Predictors	*Estimates*	*t-value; p-value*	*Estimates*	*t-value; p-value*	*Estimates*	*t-value; p-value*	*Estimates*	*t-value; p-value*
**Intercept**	0.01 (-0.06 – 0.09)	0.40; 0.689	0.19 (-0.33 – 0.70)	0.27; 0.476	0.07 (-0.01 – 0.15)	1.73; 0.084	-0.03 (-0.46 – 0.39)	-0.16; 0.872
**NPKµ**	0.02 (-0.04 – 0.08)	0.62; 0.533	0.23 (-0.25 – 0.70)	0.93; 0.354	-0.03 (-0.12 – 0.07)	-0.56; 0.574	0.23 (-0.25 – 0.71)	0.95; 0.344
**Fence**	0.00 (-0.06 – 0.06)	0.05; 0.959	0.40 (-0.09 – 0.88)	1.60; 0.109	-0.04 (-0.14 – 0.07)	-0.68; 0.495	-0.30 (-0.80 – 0.20)	-1.18; 0.238
**Intensity**	-0.00 (-0.00 – 0.00)	-0.20; 0.843	-0.00 (-0.01 – 0.00)	-0.49; 0.632	0.00 ** (0.00 – 0.00)	**3.60; 0.001**	0.00 (-0.00 – 0.00)	0.35; 0.729
**NPKµ × Fence**	-0.00 (-0.10 – 0.09)	-0.07; 0.941	-0.39 (-1.10 – 0.32)	-1.07; 0.284	-0.08 (-0.23 – 0.07)	-1.02; 0.309	0.98 * (0.23 – 1.73)	**2.56; 0.011**
**NPKµ × Intensity**	0.00 (-0.00 – 0.00)	0.07; 0.945	-0.00 (-0.01 – 0.00)	-1.28; 0.201	-0.00 ** (-0.00 – -0.00)	**-2.75; 0.006**	-0.00 (-0.00 – 0.00)	-0.17; 0.866
**Fence × Intensity**	-0.00 (-0.00 – 0.00)	-0.65; 0.513	-0.00 (-0.01 – 0.00)	-0.50; 0.619	-0.00 ** (-0.00 – -0.00)	**-2.92; 0.004**	-0.00 (-0.00 – 0.00)	-1.12; 0.263

**NPKμ × Fence × Intensity**	0.00 (-0.00 – 0.00)	0.29; 0.773	0.00 (-0.00 – 0.01)	0.86; 0.390	-0.00 (-0.00 – 0.00)	-0.86; 0.390	0.00 (-0.00 – 0.01)	1.74; 0.082
σ^2	0.04		1.94		0.10		2.36	
τ_{00}	0.12 block		0.81 block		0.09 block		0.55 block	
	0.04 site_code		0.00 site_code		0.07 site_code		0.53 site_code	
**N**	5 block		5 block		5 block		5 block	
	22 site_code		22 site_code		26 site_code		26 site_code	
**Observations**	788		788		954		954	

Table S10. Statistical output for type III ANOVA significance testing for effects of fertilization by fencing and herbivore intensity on cover and richness of key floral families. Mixed effects model results for the effects of fertilization by herbivore exclusion via fencing on Asteraceae Richness, Asteraceae Cover, Fabaceae Richness, Fabaceae Cover, Geraniaceae Richness, Geraniaceae Cover, Apiaceae Richness, Apiaceae Cover, Polygonaceae Richness, Polygonaceae Cover. The intercept is the mean value of the unfenced and unfertilized control plots. All response variables were calculated using LRRs. The alpha level for statistical significance is 0.05, and for all parameters df=1.

	**Asteraceae Richness**		**Asteraceae Cover**		**Fabaceae Richness**		**Fabaceae Cover**		**Geraniaceae Richness**		**Geraniaceae Cover**	
Predictors	*Chi-Squared Value*	*P-value*	*Chi-Squared Value*	*P-value*	*Chi-Squared Value*	*P-value*	*Chi-Squared Value*	*P-value*	*Chi-Squared Value*	*P-value*	*Chi-Squared Value*	*P-value*
**Intercept**	1.9646	0.16	0.7738	0.38	0.2696	0.63	0.0471	0.83	0.4670	0.49	0.0582	0.81
**NPKμ**	**66.1079**	**<0.0001**	**6.3481**	**0.012**	**10.0867**	**0.0015**	**4.8992**	**0.027**	0.4670	0.42	1.1241	0.29
**Fence**	6.7841	0.0092	0.0595	0.81	0.2862	0.59	0.7512	0.39	0.0767	0.78	0.6990	0.43

**Intensity**	1.0561	0.30	0.0375	0.85	0.0024	0.96	1.0575	0.30	0.0273	0.87	0.1137	0.74
**NPKμ × Fence**	**3.9660**	**0.046**	2.4674	0.12	3.8235	0.05	1.6180	0.20	0.0115	0.91	10.3716	0.0013
**NPKμ × Intensity**	0.7031	0.40	0.0650	0.80	0.3499	0.55	0.1697	0.68	0.5678	0.45	2.8045	0.09
**Fence × Intensity**	0.9460	0.33	1.0902	0.30	0.4130	0.52	0.2849	0.59	0.0307	0.86	1.9854	0.16
**NPKμ × Fence × Intensity**	**4.5719**	**0.033**	**11.8295**	**0.00058**	1.8328	0.18	0.0128	0.91	0.0325	0.86	2.6319	0.10

	**Apiaceae Richness**		**Apiaceae Cover**		**Polygonaceae Richness**		**Polygonaceae Cover**	
Predictors	*Chi-Squared Value*	*P-value*	*Chi-Squared Value*	*P-value*	*Chi-Squared Value*	*P-value*	*Chi-Squared Value*	*P-value*
**Intercept**	0.1608	0.69	0.5094	0.48	2.9977	0.08	0.0261	0.87
**NPKμ**	0.3898	0.53	0.8600	0.35	0.3162	0.57	0.8970	0.34
**Fence**	0.0026	0.96	2.5690	0.11	0.4656	0.50	1.3971	0.24
**Intensity**	0.0400	0.84	0.2367	0.63	**12.9423**	**0.00032**	0.1224	0.73
**NPKμ × Fence**	0.0055	0.94	1.1474	0.28	1.0375	0.31	**6.5526**	**0.01047**
**NPKμ × Intensity**	0.0047	0.94	1.6389	0.20	**7.5534**	**0.0060**	0.0286	0.87
**Fence × Intensity**	0.4280	0.51	0.2469	0.62	**8.5065**	**0.0035**	1.2551	0.27
**NPKμ × Fence × Intensity**	0.0830	0.77	0.7387	0.39	0.7393	0.39	3.0265	0.082

Table S11. List of forb taxonomic families present in Nutrient Network data and their within family species richness. Pretreatment data were used to generate this list. Focal families are shaded in gray and were the five most abundant families by aboveground biomass.

Family	Number of Species Present Within Family
Acanthaceae	9
Adiantaceae	2
Agavaceae	5
Alismataceae	1
Alstroemeriaceae	3
Amaranthaceae	17
Amaryllidaceae	13
Anacardiaceae	1
Apiaceae	56
Apocynaceae	15
Araceae	2
Araliaceae	2
Asparagaceae	5
Asphodelaceae	2
Aspleniaceae	1

Asteraceae (Compositae)	393
Bataceae	1
Blechnaceae	1
Boraginaceae	29
Boryaceae	2
Brassicaceae	42
Bromeliaceae	3
Cactaceae	2
Calceolariaceae	1
Campanulaceae	16
Cannabaceae	1
Caprifoliaceae	4
Caryophyllaceae	53
Chenopodiaceae	1
Cistaceae	6
Colchicaceae	1
Commelinaceae	10
Convolvulaceae	14
Cornaceae	1
Crassulaceae	9
Cucurbitaceae	1
Dennstaedtiaceae	1

Dipsacaceae	1
Droseraceae	1
Dryopteridaceae	1
Ephedraceae	1
Equisetaceae	6
Ericaceae	1
Eriocaulaceae	1
Euphorbiaceae	31
Fabaceae	197
Gentianaceae	24
Geraniaceae	21
Goodeniaceae	3
Hyacinthaceae	6
Hymenophyllaceae	1
Hypericaceae	6
Hypoxidaceae	7
Iridaceae	23
Juncaginaceae	1
Lamiaceae	32
Lentibulariaceae	1
Liliaceae	6
Linaceae	7

Loganiaceae	1
Lycopodiaceae	5
Lythraceae	3
Malvaceae	18
Mazaceae	1
Melanthiaceae	3
Melastomataceae	1
Molluginaceae	3
Montiaceae	8
Nyctaginaceae	4
Onagraceae	29
Ophioglossaceae	1
Orchidaceae	19
Orobanchaceae	10
Oxalidaceae	11
Pontederiaceae	1
Papaveraceae	3
Parnassiaceae	2
Phyllanthaceae	2
Plantaginaceae	19
Plumbaginaceae	3
Poaceae	1

Polemoniaceae	23
Polygalaceae	11
Polygonaceae	31
Polypodiaceae	1
Portulacaceae	3
Primulaceae	8
Proteaceae	1
Ranunculaceae	34
Rosaceae	58
Rubiaceae	30
Salicaceae	1
Santalaceae	3
Saxifragaceae	4
Schoepfiaceae	1
Scrophulariaceae	58
Selaginellaceae	2
Smilacaceae	1
Solanaceae	12
Thelypteridaceae	1
Thymelaeaceae	2
Ulmaceae	1
Urticaceae	2

Valerianaceae	1
Verbenaceae	7
Violaceae	19
Xanthorrhoeaceae	1
Xyridaceae	2
Zygophyllaceae	1
Family Not Given	5
