# Supplementary material for: Forb diversity globally is harmed by nutrient enrichment but can be rescued by large mammalian herbivory
Source: Commun Biol. 2025 Mar 15;8:444. doi: 10.1038/s42003-025-07882-7 (PMC11910660; doi:10.1038/s42003-025-07882-7)
Supplement: Supplementary file 3 — Description of Additional Supplementary File [file 42003_2025_7882_MOESM3_ESM.pdf]

Description Of Additional Supplementary File

File name: Supplementary Data 1
Description: Site information on metadata

File name: Supplementary Data 2
Description: Data on initial forb dominance by site

File name: Supplementary Data 3
Description: Author contributions, funding and site acknowledgements.
